# AMERICANS AND TAIWANESE: A COMPARISON OF BELIEFS IN AND PREFERENCES FOR ADVANCE DIRECTIVES BY SOCIODEMOGRAPHICS

**DOI:** 10.1093/geroni/igad104.2363

**Published:** 2023-12-21

**Authors:** Yuchi Young, Duan-Rung Chen, Ashley Shayya, Taylor Perre, Virgile Barnes, Thomas O’Grady

**Affiliations:** University at Albany, Rensselaer, New York, United States; College of Public Health, Taipei City, Taipei, Taiwan (Republic of China); University at Albany School of Public Health, Rensselaer, New York, United States; Home Care Association of New York State, Albany, New York, United States; SUNY Albany, School of Public Health, Albany, New York, United States; State University of New York at Albany, Albany, New York, United States

## Abstract

**Introduction:**

Advance directives (ADs) are legal documents that allow individuals to express their end-of-life wishes. However, cultural differences related to collectivism and family values may impact AD beliefs and preferences. This study compares AD beliefs and preferences between American and Taiwanese adults.

**Methods:**

A survey was used to collect data from adults in the US (n=166) and Taiwan (n=2000) to assess AD beliefs and preferences. Propensity score matching was used to compare the populations, and bivariate analyses were conducted to identify AD-specific differences between the two groups.

**Results:**

Compared to the US, Taiwanese participants were younger (mean age = 45 vs. 50), higher percentage female (80% vs. 63%), and higher percentage married (50% vs. 48%). Taiwanese adults had a higher belief in preparing an AD (89.3% vs. 80.7%; p=0.02) and were more willing to complete one than American adults (85.6% vs. 63.1%; p< 0.01). 59.4% of Taiwanese adults preferred their family/loved ones make healthcare decisions, compared to 46.0% of Americans (p=0.01). However, only 50.8% of Taiwanese adults believed their loved ones’ decisions aligned with their wishes, while 79.5% of Americans held this belief (p< 0.001).

**Conclusion:**

This study reveals noteworthy differences in AD beliefs and preferences between the two groups, with Taiwanese adults valuing AD preparation more and being more willing to complete them. Taiwanese adults also rely on their loved ones to make AD decisions, even though they believe these wishes may not align with their own. These differences likely stem from cultural norms of filial piety and collectivism in Taiwan.

